# The severity of hereditary porphyria is modulated by the porphyrin exporter and Lan antigen ABCB6

**DOI:** 10.1038/ncomms12353

**Published:** 2016-08-10

**Authors:** Yu Fukuda, Pak Leng Cheong, John Lynch, Cheryl Brighton, Sharon Frase, Vasileios Kargas, Evadnie Rampersaud, Yao Wang, Vijay G. Sankaran, Bing Yu, Paul A. Ney, Mitchell J. Weiss, Peter Vogel, Peter J. Bond, Robert C. Ford, Ronald J. Trent, John D. Schuetz

**Affiliations:** 1 Department of Pharmaceutical Sciences, St Jude Children's Research Hospital, Memphis, Tennessee 38105, USA; 2 Department of Medical Genomics, Royal Prince Alfred Hospital, Sydney, New South Wales 2050, Australia; 3 Sydney Medical School, University of Sydney, Sydney, New South Wales 2006, Australia; 4 Department of Tissue Cell Biology, St Jude Children's Research Hospital, Memphis, Tennessee 38105, USA; 5 Department of Structural Biology, Faculty of Life Sciences, University of Manchester, Manchester M13 9PT, UK; 6 Department of Computational Biology, St Jude Children's Research Hospital, Memphis, Tennessee 38105, USA; 7 Department of Hematology, St Jude Children's Research Hospital, Memphis, Tennessee 38105, USA; 8 Division of Hematology/Oncology, Boston Children's Hospital, Boston, Massachusetts 02115, USA; 9 New York Blood Center, New York, New York 10065, USA; 10 Department of Pathology, St Jude Children's Research Hospital, Memphis, Tennessee 38105, USA; 11 Bioinformatics Institute, 30 Biopolis Street, Singapore 138671, Singapore; 12 Department of Biological Sciences, National University of Singapore, 14 Science Drive 4, Singapore 117543, Singapore

## Abstract

Hereditary porphyrias are caused by mutations in genes that encode haem biosynthetic enzymes with resultant buildup of cytotoxic metabolic porphyrin intermediates. A long-standing open question is why the same causal porphyria mutations exhibit widely variable penetrance and expressivity in different individuals. Here we show that severely affected porphyria patients harbour variant alleles in the *ABCB6* gene, also known as *Lan*, which encodes an ATP-binding cassette (ABC) transporter. Plasma membrane ABCB6 exports a variety of disease-related porphyrins. Functional studies show that most of these *ABCB6* variants are expressed poorly and/or have impaired function. Accordingly, homozygous disruption of the *Abcb6* gene in mice exacerbates porphyria phenotypes in the *Fech*^*m1Pas*^ mouse model, as evidenced by increased porphyrin accumulation, and marked liver injury. Collectively, these studies support *ABCB6* role as a genetic modifier of porphyria and suggest that porphyrin-inducing drugs may produce excessive toxicities in individuals with the rare Lan(−) blood type.

## Abstract (web summary)

Accumulation of intermediates of haem biosynthesis, porphyrins, is harmful and usually inherited, but it is unclear how the same mutation may make some individuals more ill than others. Here, the authors show that a porphyrin transporter ABCB6 is a modulator of porphyria, and that patients with functionally defective ABCB6 show more severe symptoms.

## Graphical abstract

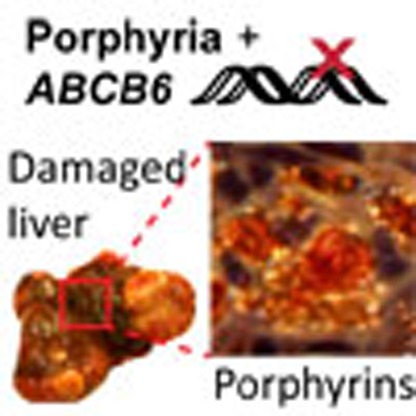

The inherited porphyrias are metabolic disorders of haem biosynthesis caused by mutations in genes coding for enzymes in the haem biosynthetic pathway^1^,^2^. The intermediates of haem biosynthesis are toxic and their accumulation is particularly damaging to the liver, haematopoietic system, skin and neural tissues, accounting for the characteristic clinical symptoms. Most porphyrias are inherited as autosomal dominant mutations with marked variability in phenotypes that cannot be accounted for by the causal mutation. Indeed, a long-standing clinical mystery has been why some porphyria patients become more ill than others, even within the same pedigree^3^,^4^. We hypothesized that the variable penetrance and expressivity of porphyrias might be due to genetic modifiers^3^,^5^,^6^,^7^.

It is unknown if porphyrin transporters modify the severity of porphyria, especially those transporters that are expressed in red blood cells where most of haem biosynthesis occurs. Multiple porphyrins are exported from differentiating red blood cell model systems, but the identity of the transporter(s) in normal red blood cells remains unknown^8^,^9^. We hypothesized that porphyrin transporters might impact porphyria. Disruptions in porphyrin export might produce overaccumulation of toxic porphyrins (protoporphyrin IX (PPIX), coproporphyrins (CPs) and uroporphyrins (Uros))^10^ in red blood cells. The export of PPIX from red blood cells is mediated by the plasma membrane transporter ABCG2 (refs 11, 12, 13, 14), but it is unknown if this is the sole energy-dependent exporter of porphyrins. Another porphyrin transporter, ABCB6, was recently identified as the Lan blood group antigen in the plasma membrane of red blood cells^15^, but its function in the red cells as a porphyrin exporter has not been directly tested.

Here we show that variant alleles of *ABCB6* gene that are identified in individuals with severe cases of porphyrias have poor expression and/or are defective. We also demonstrate that ABCB6 mediates the plasma membrane export of porphyrins in red cells. Imposing *Abcb6* deletion on the *Fech*^*m1Pas*^ mouse model exacerbates porphyria symptoms, as evidenced by increased red blood cell and hepatic porphyrin concentrations, and enhanced liver injury. These studies show that rare *ABCB6* variant alleles modify the severity of porphyria symptoms in patients. More generally, we show how complementary clinical observations, genetic and biochemical studies synergize to inform precision medicine.

## Results

### WES reveals *ABCB6* variants in severely affected patients

**Figure 1:**
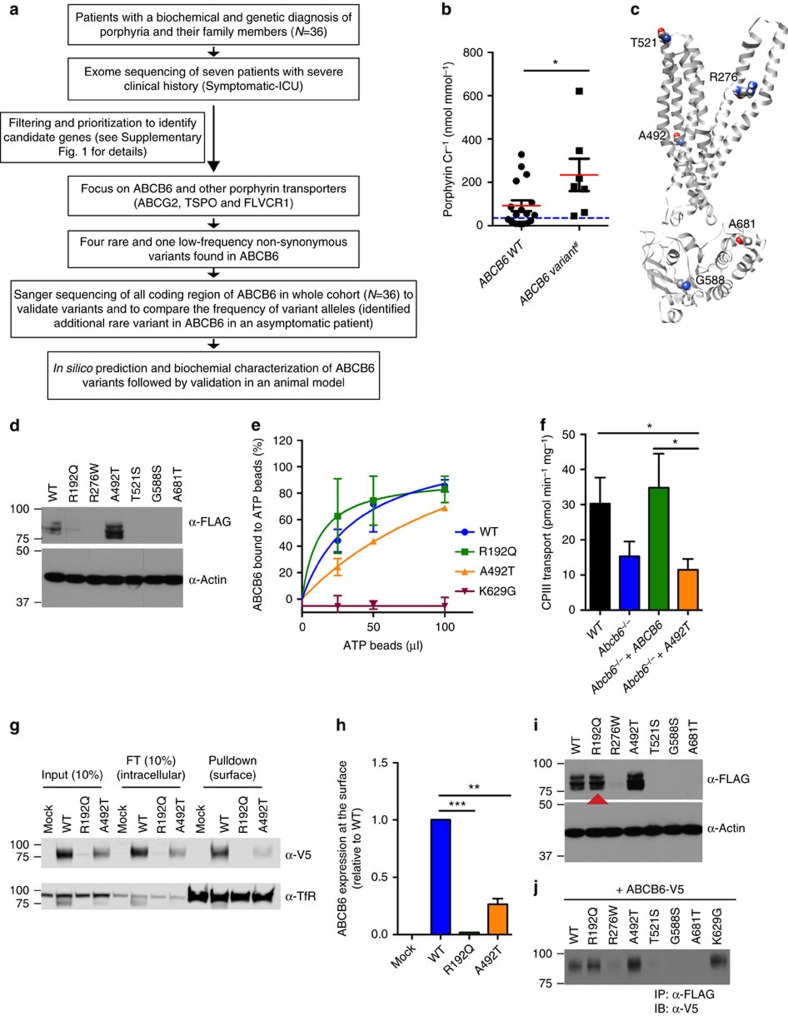
Clinical and biochemical analyses of rare variant alleles of *ABCB*6 in porphyric patients. (**a**) Flow chart describing strategies to identify variants associated with severe porphyria symptoms. (**b**) Urinary porphyrin levels normalized to creatinine. ^#^Patients with only defective *ABCB6* alleles were included. The asymptomatic patient with *ABCB6* R192Q variant allele showed a low porphyrin levels compared with patients with other *ABCB6* variant alleles. *n* for WT and variant are 17 and 7, respectively. (**c**) Ribbon diagram showing the location of amino acids encoded by rare variant *ABCB6* alleles (for clarity, only a monomer is shown). The variant R192Q is not shown because high-resolution structural data are not available as this residue lies in a non-conserved region among ABC transporters. (**d**) Immunoblot of transient transfection of ABCB6 wild-type (WT) and variant alleles (the epitope tag was previously shown to not disrupt function^18^). (**e**) The ATP-binding capacity measured from ATP-agarose beads pull-down was plotted (*n*=2). (**f**) ATP-dependent CPIII transport into membrane vesicles prepared from indicated MEL cell lines is shown (*n*=2). (**g**) ABCB6 WT and variant expression at the cell surface in *Abcb6*^−/−^ mouse embryonic fibroblast was determined by cell surface biotinylation assays. FT, flow through. (**h**) ABCB6 pulled down by streptavidin-agarose beads was quantified by densitometry. Two independent experiments were performed. Only the representative results shown. (**i**) Immunoblot of ABCB6 WT and variants when co-transfected with V5-tagged WT ABCB6. The red arrow shows R192Q is stabilized by WT ABCB6. Two independent experiments were performed. Only the representative results shown. (**j**) Co-immunoprecipitation assay to assess interaction between ABCB6 WT and variant alleles. *P<0.05; **P<0.01; ***P<0.001 using Student *t*-test with error bars showing s.d.

At the Royal Prince Alfred Hospital in Australia, a cohort of 36 porphyria patients of European descent and their family members were evaluated for clinical history, biochemical profile and detection of pathogenic mutations in haem biosynthetic pathway genes (*HMBS*, *CPOX*, *PPOX* or *FECH*; Supplementary Table 1). We performed whole-exome sequencing (WES) on seven individuals with the most severe clinical phenotypes (symptomatic intensive care unit (ICU); for patient stratification, see Supplementary Methods; Fig. 1a, Supplementary Fig. 1a). The WES data of these individuals were filtered to develop a prioritized candidate gene list, then enrichment analysis was performed using Database of Annotation, Visualization and Integrated Discovery (DAVID)^16^,^17^ (see Methods; Supplementary Fig. 1a; Supplementary Table 2). The ‘ABC transporters' cluster defined by KEGG_PATHWAY and INTERPRO was significantly enriched among the candidate genes (Supplementary Table 3; enrichment score=1.93, false discovery rate *P*<0.20). ABC transporters mostly consist of four domains, two transmembrane domains, each composed of six membrane-spanning helices, and two nucleotide-binding domains (NBDs). While the NBDs are highly conserved, the transmembrane domain are diverse, a likely reflection of the wide variety of substrates. This ABC cluster cluster included two porphyrin transporters, ABCG2 and ABCB6 (refs 11, 12, 18). ABCG2 mediates PPIX export from red blood cells, the major site of haem synthesis^11^,^12^. Plasma membrane ABCB6 defines the Langereis (Lan) antigen^15^, which can cause severe haemolytic transfusion reactions and haemolytic disease of the fetus and newborn^19^. Lan(−) individuals are typically asymptomatic, thus the physiological function of plasma membrane ABCB6 is unknown.

Porphyrins are exported from erythroid and liver cells, presumably to reduce cytotoxicity and/or membrane damage during massive haem synthesis^8^,^9^. Therefore, we focused on porphyrin transporter genes *ABCB6*, *ABCG2*, *TSPO*^20^ and *FLVCR1* (refs 13, 14). In the severely affected porphyria patients, five heterozygous rare (minor allele frequency (MAF) <1%) and one heterozygous low-frequency (MAF <5%) variants in *ABCB6* were identified, and the unimpaired *ABCG2* V12M variant^21^ was found in four patients (Table 1; Supplementary Table 1). WES did not identify any rare or low-frequency variants in *TSPO* and *FLVCR1.* We then analysed *ABCB6* and *ABCG2* V12M in our entire porphyria patient cohort by Sanger sequencing. The frequency of *ABCG2* V12M variant in the severely affected individuals was not significant compared with the rest of the cohort (Fisher's exact test, *P*=0.076, two sided).

Overall, 6/7 (85%) of the severely affected patients were heterozygous for *ABCB6* variants. ICU admission rate was 7.1% for porphyria patients with homozygous wild-type (WT) *ABCB6* alleles versus 62.5% for those with heterozygous rare or low-frequency non-synonymous *ABCB*6 alleles (Fisher's exact test, *P*=0.0026). The urinary porphyrin concentrations were almost 2-fold higher (*P*=0.029) in patients harbouring *ABCB6* variant alleles versus patients with WT *ABCB6*, and 5.9-fold higher than individuals without porphyria^22^ (Fig. 1b). The higher frequency of these *ABCB6* variants in these severely affected porphyria patients of European descent compared with European Americans tabulated in the NHLBI Exome Variant Server (22.2% versus 3.8%, Fisher's exact test, *P*=0.0034, two sided; http://evs.gs.washington.edu/EVS/; Supplementary Table 4) suggests these variants might be non-functional.

### Variant ABCB6 alleles are non-functional

ABCB6 is a half transporter composed of a single membrane-spanning domain (MSD) and a cytoplasmic NBD^23^ that functions as a homodimer^18^,^24^. In Fig. 1c, the variant residues are highlighted on a homology model of ABCB6 (Supplementary Fig. 1b) with the ABCB6 monomer shown for clarity. The substitutions at R276W and A492T are in the MSDs, whereas T521S is in an extracellular loop, and G588S and A681T are near the NBD. *In silico* analyses^25^ predicted that R276W and A681T are damaging variants (Supplementary Table 5). However, these computational approaches are not sensitive or specific^26^, but our homology models for ABCB6, in both the inward-facing conformation (Supplementary Fig. 5, PDB ID 3ZDQ) and the outward-facing conformation (a model based on Sav1866 (PDB ID 2HYD)), suggest that the interaction between R276 and D397 would be disrupted with the latter exposed to the hydrophobic portion of the lipid bilayer (a thermodynamically unfavourable location). Hence, we carried out functional studies to validate the significance of the variants.

There was minimal or no difference in translation of the WT or ABCB6 variants (Supplementary Fig. 2a). However, the R276W, T521S, G588S, A681T and R192Q variants (FLAG tagged) were expressed either poorly or undetected when transiently expressed (Fig. 1d).

We examined the effects of the only mutations that produced detectable levels of ABCB6 (R192Q and A492T) on ATP and substrate (haem) binding. Protein extracts prepared from NIH3T3 cells expressing V5-tagged ABCB6 WT, R192Q, A492T or K629G (a non-ATP-binding mutant^18^) were incubated with ATP- or hemin-agarose beads, and the amount of ABCB6 bound to the beads was determined. Both R192Q and A492T were capable of binding haem (Supplementary Fig. 2b,c). ABCB6 R192Q-bound ATP-agarose normally, while the A492T-substituted protein displayed markedly impaired ATP binding and as expected the positive control K629G did not bind ATP (Fig. 1e).

To investigate A492T function, we used CRSPR-Cas9 technology^27^ to create *Abcb6* deficiency in an erythroid cell line (murine erythroleukaemia (MEL) cells^28^; Supplementary Fig. 2d). A membrane vesicle transport assay was established that demonstrated maximal ATP-dependent CPIII transport in ABCB6 proficient cells (Supplementary Methods). To directly compare A492T with WT, both complementary DNAs were subcloned into a retroviral expression vector that harboured a downstream ribosomal entry site followed by a green fluorescent protein (GFP) to allow selection. The A492T and WT retroviruses were transduced into the *Abcb6*-null cells and membrane vesicles prepared. Despite similar protein levels (Supplementary Fig. 2d) in the membrane vesicles, A492T was incapable of transport (Fig. 1f). *Abcb6*-null cells were then used to determine the plasma membrane expression of A492T and R192Q using surface biotinylation labelling^29^ (Fig. 1g). The surface expression of A492T was only 26% of WT and R192 was barely detectable (Fig. 1h). In total, reduced membrane expression and defective ATP-binding appear to account for A492T loss of function.

Because these porphyria patients are heterozygous for the variant alleles, we co-expressed each FLAG-tagged variant with V5-tagged WT *ABCB6* (Fig. 1i). WT ABCB6 rescued expression of the R192Q protein. In contrast, by immunoprecipitation with the anti-FLAG, WT ABCB6 expression was reduced by variants R276W, T521S and G588S, indicating that these behave as dominant-negative proteins (Fig. 1j). The near-normal ATP- and hemin binding of R192Q, in the presence of WT ABCB6, are consistent with this individual being asymptomatic and normal urinary porphyrin level (9 nmol mmol^−1^ per porphyrin creatinine).

### Porphyrin exporter ABCB6 modifies porphyria phenotypes

PPIX, CP and/or Uro levels are elevated in the urine and faeces of porphyria patients^1^,^2^. Bone marrow transplantation studies cured erythropoietic protoporphyria^30^, indicating that most of these porphyrins are derived from haematopoietic cells, specifically red blood cells, where >85% of haem synthesis occurs. Early studies noted that both CPs and Uros were exported from erythroid cell lines via unknown mechanisms^8^,^9^.

**Figure 2:**
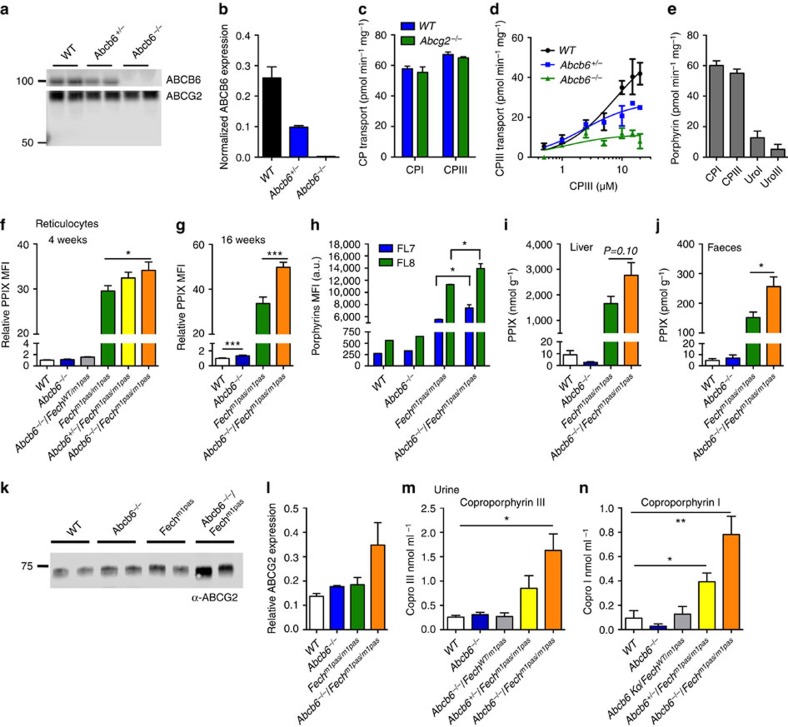
ABCB6 is a broad porphyrin transporter that affects porphyrin accumulation. (**a**) Immunoblot of red blood cell membrane and (**b**) its quantification shows ABCB6 levels in the membrane (*n*=2). (**c**) Red blood cell (RBC) membrane samples prepared from wild-type (WT) or *Abcg2*^−*/*−^mice were assayed for ATP-dependent CPI and CPIII transport (*n*=2). (**d**) RBC membranes prepared from indicated genotypes of mice were assessed for an ATP-dependent transport of CPIII to estimate *K*_M_ and *V*_max_ (*n*=2). (**e**) An ATP-dependent transport of CPI, CPIII, UroI and UroIII was measured in WT RBC membranes (*n*=2). PPIX levels in reticulocytes (identified as thiazole orange (TO)^+^/Ter119^+^) from mice with different genetic compositions at (**f**) 4 weeks and (**g**) 16 weeks were analysed by flow cytometry. MFI, mean fluorescence intensity. Each point represents 6–19 animals and error bars show s.e.m. (**h**) Intracellular PPIX (FL8) and porphyrins (FL7) in reticulocytes (TO^+^/Ter119^+^) from mice transplanted with haematopoietic progenitors from indicated genotypes of mice were measured by flow cytometry (up to six mice in each group was used). High-performance liquid chromatography (HPLC) determination of PPIX in the liver (*n*=5 and 6) from 4-week-old mice (**i**) and in (**j**) faecal samples collected from mice (*n*=4–10). (**k**) Immunoblot of liver samples from indicated genotypes of mice for ABCG2 expression and (**l**) its quantification (*n*=2). Urine samples from 4-week-old mice were analysed for (**m**) CPIII (*n*=3–7) and (**n**) CPI concentration using HPLC (*n*=3–6). *P<0.05; **P<0.01; ***P<0.001 using Student's *t*-test with error bars showing s.d., unless otherwise indicated.

Candidate proteins expressed in erythroid cells include FLVCR, ABCB6 and ABCG2. We investigated red blood cell membrane expression of ABCG2. Insufficiency or deficiency of ABCB6 was not associated with increased ABCG2 protein (Fig. 2a,b). While ABCG2 exports PPIX (refs 11, 12, 21), it is unknown if it exports other porphyrins. We prepared red cell plasma membrane ghosts from *Abcg2*-null and WT animals to assess transport of other porphyrins. Unexpectedly, *Abcg2* loss had no effect on CP transport (Fig. 2c). To investigate whether ABCB6 was a physiologically important porphyrin exporter, reticulocytes from WT or *Abcb6*^−*/*−^ mice were incubated with PPIX and/or fumitremogin C, a specific ABCG2 inhibitor. ABCB6 and ABCG2 appear to have additive effects with the strongest PPIX accumulation occurring when both ABCB6 was absent and ABCG2 was inhibited (Supplementary Fig. 3). Next, direct membrane transport of CPs by ABCB6 was measured using red cell ghosts prepared from WT and gene-ablated mice (Fig. 2d). Loss of ABCB6 strongly reduced CPIII transport in a dose-dependent manner (Fig. 2d), with over 75% of total CPIII transport in red cell membranes accounted for by ABCB6 (*V*_max_: *WT* 59.4, *Abcb6*^*+/*−^ 28.13 and *Abcb6*^−*/*−^ 11.51 pmol min^−1^ mg^−1^). Furthermore, ABCB6 also readily transported CPI, UroI and UroIII (Fig. 2e). Thus, ABCB6 is the predominant CPIII exporter, at least in murine red blood cells.

To investigate the role of *Abcb6* as a modifier of porphyria, we used the ferrochelatase-deficient mouse (*Fech*^*m1Pas*^)^31^ and intercrossed single mutant strains that had first been backcrossed into the BALB/c background^31^,^32^. In accord with our genetic findings and biochemical studies, reticulocyte PPIX concentrations in *Fech*^*m1Pas*^ were enhanced by *Abcb6* loss in a dosage-dependent manner in 4-week-old mice (Fig. 2f). By 16 weeks, the loss of both *Abcb6* alleles increased PPIX concentrations by ∼50% in reticulocytes of *Fech*^*m1Pas*^ mice (Fig. 2g).

The capacity of ABCB6 to transport multiple porphyrins suggests that its deficiency in erythroid cells might account for the elevated porphyrins in *Abcb6*^−*/*−^/*Fech*^*m1Pas*^ mice. To investigate this, we transplanted haematopoietic stem cells and progenitors from *Abcb6*^−*/*−^ only, *Fech*^*m1Pas*^ only and *Abcb6*^−*/*−^/*Fech*^*m1Pas*^ mice into lethally irradiated congenic WT recipients and assessed porphyrin levels in red blood cells using fluorescence emission filter sets to selectively measure PPIX (FL8) and all other porphyrins (FL7; Fig. 2h). In accord with the porphyrin transport capability of ABCB6, both PPIX and other porphyrins were significantly higher in red blood cells derived from mice transplanted with *Abcb6*^−*/*−^/*Fech*^*m1Pas*^ progenitors, indicating red blood cell-derived porphyrins likely account for the elevated porphyrins in *Abcb6*^−*/*−^/*Fech*^*m1Pas*^ mice.

In *Fech*^*m1Pas*^ mice, the loss of *Abcb6* increased hepatic PPIX by 60% (Fig. 2i). Because the magnitude of liver PPIX elevation in the composite *Abcb6*^−*/*−^ and *Fech*^*m1Pas*^ animals did not directly correspond with the elevated PPIX concentration found in reticulocytes, we determined faecal concentrations of PPIX. The amount of PPIX in the faeces of *Abcb6*^−*/*−^/*Fech*^*m1Pas*^ animals was 64% higher than in the *Fech*^*m1Pas*^ animals (Fig. 2j). The hepatic concentration of PPIX might have been even higher if not for the compensatory increase in hepatic expression of the canalicular-localized ABCG2 (ref. 33; Fig. 2k,l). In addition, the urinary concentration of CPs in *Fech*^*m1Pas*^ mice was greatest when both *Abcb6* alleles were absent and modestly increased by the loss of one *Abcb6* allele (Fig. 2m,n). Thus, insufficiency of *Abcb6* increases the porphyrin concentrations in tissues and excreta in a mouse model for porphyria.

**Figure 3:**
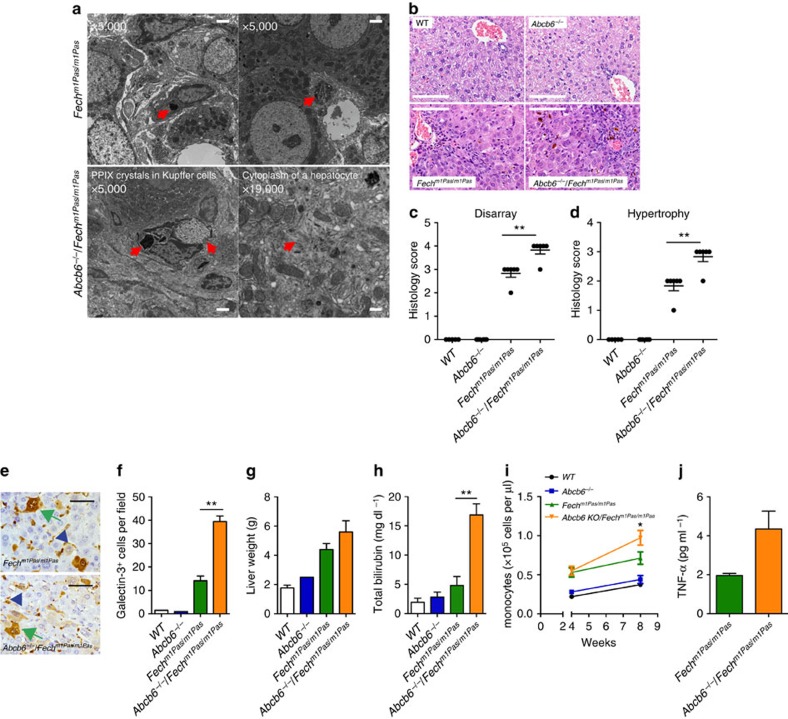
Loss of ABCB6 worsens liver injuries in *Fech*^*m1Pas*^ mice. (**a**) Electron micrographs of liver specimens from *Fech*^*m1Pas*^ and *Abcb6*^−*/*−^/*Fech*^*m1Pas*^ mice. The red arrows indicate PPIX crystals. Scale bars, 2 μm (for × 5,000 images); 500 nm (for × 19,000 image). (**b**) Haematoxylin- and eosin-stained liver sections were blindly scored for (**c**) liver organization (disarray) and (**d**) hypertrophy using the scale: 0=normal, 1=minimal, 2=mild, 3=moderate, 4=marked and 5=severe. Each dot represents an individual animal (*n*=5). Scale bars, 100 μm. (**e**) Galectin-3 staining of liver sections showing reactive Kupffer cells (blue arrowhead) and hepatocytes (green arrowhead) Scale bars, 50 μm. (**f**) Galectin-3-positive hepatocytes were quantified (average of 10 fields from a slide, *n*=3 each). (**g**) Liver weight (up to three samples) and (**h**) serum total bilirubin (up to eight samples) from indicated genotypes of mice. (**i**) Monocyte counts were determined from complete blood count values (*n*=13–25 samples. Error bars show s.e.m.). (**j**) Inflammatory cytokine levels in the serum were determined by enzyme-linked immunosorbent assay (*n*=2 and 3, respectively). *P<0.05; **P<0.01 using Student's *t*-test with error bars showing s.d., unless otherwise indicated.

We also investigated whether the loss of *Abcb6* exacerbates liver damage caused by *Fech* deficiency. Liver histologies from 4-week-old WT, *Abcb6*^−*/*−^, *Fech*^*m1Pas*^ and *Abcb6*^−*/*−^/*Fech*^*m1Pas*^ mice were analysed by light and transmission electron microscopy (Fig. 3). Electron microscopy showed extensive crystalline PPIX deposits in the cytosol of hepatocytes from *Abcb6*^−*/*−^/*Fech*^*m1Pas*^ mice (Fig. 3a), in agreement with the elevated liver PPIX concentrations (Fig. 2i). The *Abcb6*^−*/*−^/*Fech*^*m1Pas*^ mice also exhibited increased hepatocyte hypertrophy and enhanced parenchymal disarray (Fig. 3b–d). Galectin-3 staining, a marker for liver injury, was strikingly increased in hepatocytes from *Abcb6*^−*/*−^/*Fech*^*m1Pas*^ mice compared with those from *Fech*^*m1Pas*^ mice (Fig. 3e,f). The *Abcb6*^−*/*−^/*Fech*^*m1Pas*^ mice also exhibited larger livers and significantly higher total serum bilirubin levels (Fig. 3g,h). Consistent with Kupffer hypertrophy, circulating monocytes (Fig. 3i) and levels of the inflammatory cytokine tumour necrosis factor-α were increased in *Abcb6*^−*/*−^/*Fech*^*m1Pas*^ mice (Fig. 3j).

## Discussion

Although elevated porphyrins are a hallmark of multiple types of porphyria, the role of porphyrin transporters (TSPO, ABCG2, ABCB6 and FLVCR1) as modulators of disease severity has not been investigated. Using deep sequencing, biochemical analysis and a new mouse model, we show here that ABCB6 is a genetic modifier of porphyria that mitigates its severity by expelling porphyrins. A previous report showed that ABCB6 was expressed at the red blood cell membrane, but did not uncover a function^15^. This was probably because, among healthy non-porphyric individuals, *ABCB6* deficiency *per se* would not result in excess porphyrin accumulation, although ABCB6 acts as a ‘broad spectrum' exporter of multiple porphyrins at the plasma membrane. Accordingly, the impact of *ABCB6* deficiency emerges only when porphyrins accumulate during disrupted haem biosynthesis. Although the current porphyria cohort is small, this study indicates that among individuals with porphyrias, functionally defective *ABCB6* alleles are strongly associated with disease intensification, providing new mechanistic insights into the genetics and biochemistry of porphyrias.

By stimulating haem synthesis and porphyrin production, a wide array of drugs can exacerbate porphyria symptoms in patients (http://www.porphyriafoundation.com/drug-database). It is possible that these drugs could elicit porphyria-like symptoms in Lan(−) individuals with normal haem synthetic enzyme genes. Moreover, drugs that also interfere with ABCB6 function might produce similar symptoms. In support of this proposition is our finding that mice lacking one copy of *Abcb6* have increased porphyrins. Thus, unanticipated drug or xenobiotic-induced porphyrias might be especially prevalent among Lan(−) individuals.

## Methods

### Patient cohort

Patients with a biochemical and clinical diagnosis of porphyria and their family members were recruited from the porphyria clinic at the Royal Prince Alfred Hospital, Sydney, Australia. A total of 36 patients from 26 families were recruited. The study was approved by the Sydney Local Health District Ethics Review Committee and written informed consent was obtained from all participants. As porphyria is a rare disease and the available patients were limited, statistical methods were not used in advance to determine the sample size. Nonetheless, statistical analysis of the porphyria patients harbouring the WT ABCB6 allele indicated that these were normally distributed (D'Agostino and Pearson omnibus normality test). Causative porphyria mutations were identified by Sanger sequencing in all 36 patients. The severity of porphyria was categorized into four groups based on clinical history: (1) symptomatic ICU: patients who have had life-threatening porphyria attacks requiring ICU admission and administration of haem arginate. A patient with erythropoietic protoporphyria (X1) who developed fatal liver failure was also included in this group; (2) symptomatic admission: patients who have had biochemically confirmed porphyria attacks and required hospital admission, but did not require ICU admission; (3) symptomatic no admission: patients who have had symptoms consistent with a porphyria attack, but no biochemical confirmation and never required hospital admission; and (4) asymptomatic: patients who never had symptoms consistent with a porphyria attack.

### Porphyrin measurement from patients

Porphyrin biochemistry was performed by the Department of Clinical Chemistry at the Royal Prince Alfred Hospital, Sydney, Australia. Some historical results were not available. Urine total porphyrin was measured by fluorescence in the presence of weak hydrochloric acid against a standard of known concentrations in a Perkin Elmer LS50B spectrofluorimeter (*λ*_em_ 596 nm, slit width 16 nm band pass, *λ*_ex_ 380–440 nm, slit width 6 nm band pass).

### Exome sequencing

A total of seven symptomatic ICU patients were identified (B7, C1, F1, I1, J1, T1 and X1—see Supplementary Table 1). All symptomatic ICU patients had exome sequencing to look for modifier genes. The exome-sequencing data have been deposited in European Nucleotide Archive under accession code PRJEB14229 (http://www.ebi.ac.uk/ena/data/view/PRJEB14229). Barcoded 75–35 bp paired-end fragment libraries were constructed from 2 μg of genomic DNA using a Covaris focused-ultrasonicator (Covaris, Wodburn, MA) and AB Library Builder System (Life Technologies, Carlsbad, CA). Exomes were captured using the TargetSeq exome enrichment kit (Life Technologies) according to the manufacturer's protocol. Captured libraries were quantified by TaqMan assay, amplified by emulsion PCR and enriched using the SOLiD EZ Bead system (Life Technologies). Sequencing was performed on the SOLiD 5500xl platform (Life Technologies). Mean percentage of coverage ≥5 × was 94.3% with an average depth of coverage of × 60.2.

Mapping and variant calling of exome sequence reads were performed on LifeScope Genome Analysis Software (Life Technologies) using the Genome Reference Consortium human reference sequence (GRCh37) as reference. The output GFF3 files were annotated using wANNOVAR^34^. Annotated variants were uploaded onto Galaxy^35^,^36^,^37^ and filtered for coverage (≥5), allelic imbalance (variant/reference read starts ratio between 0.3 and 3.0), read quality score (≥25) and population frequency (≤5%). Variants in candidate genes within the haem biosynthetic pathway and the four porphyrin transporters (*TSPO*, *FLVCR1*, *ABCB6* and *ABCG2*) were manually reviewed. Rare and low-frequency variants identified in these genes detected by exome sequencing were confirmed by Sanger sequencing. Sanger sequencing of *ABCB6* was also performed for the whole cohort to exclude the presence of rare *ABCB6* variants in other patients.

### Filtering strategy for exome-sequencing data

Raw sequence data were filtered as shown in Supplementary Fig. 1. During the first stage of technical filtering, only variants with raw coverage ≥5, novel read starts ≥3, high base quality scores (QV) ≥25 and where Reference/Novel read start ratio was between 0.3 and 3.0 (an indicator of allelic imbalance and presence of clonal reads) were passed through. Systematic false positives were removed using data generated on the same platform from 39 control exomes of unrelated white European individuals not known to have porphyria. In addition, any potential sequencing platform-based systematic error was removed. Variants that passed the technical filtering stage were further filtered for biological plausibility using population-based estimates of frequency and bioinformatics predictions of the functional impact of the variant. Only non-synonymous variants and those residing in the acceptor/donor splice sites were further evaluated. Variants with MAF >0.05 in control samples from the NHLBI-ESP6500 project were considered common in the general population and not likely to be pathogenic. We also used the Residual Variation Intolerance Score to rank the tolerance of the gene to missense, nonsense or acceptor/donor splice site variants^38^. Residual Variation Intolerance Score ≤1 were prioritized for this study.

### Selection of candidate genes from WES data

Prioritized variants from WES filtering were annotated to candidate genes. Genes with ≥3 variants detected among the seven WES data set were uploaded into the DAVID^16^,^17^ for pathway analysis and enrichment of functional categories (for example, gene ontology terms).

### Homology modelling

The functional unit of ABCB6 is a homodimer that localizes to both the outer mitochondrial membrane^18^,^39^,^40^,^41^ and the plasma membrane of cells^15^,^39^. Because ABC transporter substrates typically interact with the MSDs, homology models of ABCB6 were developed based on the outward-facing structure of the homodimeric ABC transporter, Sav1866 (ref. 42), and the inward-facing structure of the homodimeric ABCB10 (refs 43, 44; Supplementary Figs 4 and 5). Outward- and inward-facing models for ABCB6 were built using the Sav1866 structure (PDB ID 2HYD) and the ABCB10 structure (PDB ID 3ZDQ). The model starts with residue L248 (excluding R192 from the analysis) and ends close to the C terminus at G827. Preliminary sequence alignment was carried out with CLUSTALW and the most conserved region with fewest gaps (248–827) was selected for alignment against Sav1866 (residues 1–578). All the necessary modifications of the PDB file were performed utilizing the PDB cleaner routine^45^. The alignment was then imported into the Modeller 9.11 program^46^ for the generation of 100 structural models. The models were structurally aligned against the template structure and the best was used for the generation of a dimeric complex. Energy minimization of the homodimer was carried out using MMTK^47^ implemented within the Chimera software suite^48^ with 1,000 steps of steepest descent and of step size 0.002 nm to remove significant clashes, followed by 10 steps of conjugate gradient minimization (step size 0.002 nm) with no atoms fixed, hydrogen atoms included and charged residues assigned. The quality of the models was monitored at intervals during the minimization procedure using the Procheck program^49^, and a final model was selected in terms of clashes and stereochemistry. Visual inspection of the model was also used as a check for its accuracy, especially for the relatively poorly conserved transmembrane regions. If correctly aligned, the model would be expected to display a 4 nm thick band of hydrophobic residues exposed to the lipid bilayer with few or no lipid-exposed charged residues. Such a band is clearly observed. The model offers no clues as to the location of the N-terminal membrane-spanning region of ABCB6 (which shows no homology with known ABC protein structures). No patch of polar or charged residues on the surface of the model could be identified that might point to the packing of additional transmembrane helices at such a position.

### Biochemical studies

Surface biotinylation, immunoblotting, immunoprecipitation and hemin-agarose pull-down assays using transiently transfected NIH3T3 cells (ATCC CRL-1658) were performed as described below^18^,^41^. Twenty-four hours after transient transfection, cells were collected, lysed and subjected to hemin-agarose binding or ATP-agarose binding at room temperature for 30 min. ATP-agarose beads were obtained from Sigma-Aldrich (St Louis, MO) and binding assays were performed at room temperature. TnT T7 Coupled Reticulocyte Lysate System was used for *in vitro* translation of ABCB6 variant alleles from T7 promoter (Promega, Madison, WI).

### *Fech^m1Pas^* mouse model and analysis

All experiments involving mice were reviewed and approved by the Institutional Animal Care and Use Committee at St Jude Children's Research Hospital (SJCRH). C.Cg-*Fech*^*m1Pas*^/J mice (#002662) were obtained from Jackson Laboratory (Bar Harbor, ME) and BALB/cByJ (#001026) mice were used as WT control. *Abcb6*^−*/*−^ mice^41^ were backcrossed to BALB/cByJ strain and the Illumina Mouse MD Linkage Chip containing 1,449 single-nucleotide polymorphism loci was used to confirm that the strains were ∼99% identical. Reticulocyte PPIX levels were measured by fluorescence-activated cell sorting (FACS) and complete blood counts measured at Veterinary Pathology Core (SJCRH). Adult (>3-month old) male and female mice were used for the study unless specified otherwise in the text.

Lineage-negative haematopoietic progenitors (CD4^−^, CD8^−^, Mac1^−^, Gr1^−^, B220^−^, NK1.1^−^ and Ter119^−^) were isolated from adult female mice from each genotype using MACS Miltenyi Biotec (San Diego, CA, USA) depletion. The progenitors were allowed to recover overnight and injected into lethally irradiated congenic recipient mice. Reticulocyte PPIX levels were analysed starting 5 weeks post transplantation.

The mice were perfused with PBS containing glutaraldehyde and further processed for electron microscopic analysis of the liver. The tissues were fixed in formalin and paraffin-embedded sections were prepared for histological analysis.

### PPIX and haem measurement

Intracellular PPIX levels were determined by flow cytometric analysis^18^. Where indicated, two emission filter sets, 670/30 and 610/20, were used to distinguish PPIX from total cellular porphyrins, respectively. Following acidic acetone extraction, tissue, urinary and faecal porphyrins, and haem were measured by high-performance liquid chromatography^41^.

### CRISPR/Cas9-mediated Abcb6 deletion in MEL cells

CRISPR/Cas9 technology was used to disrupt *Abcb6* gene in murine erythroleukaemia cells. MEL cells were transduced with pCMV-Cas9-RFP plasmid containing specific guide RNA sequence (tactgcgagaccgaagggccgg) targeted to *Abcb6* (purchased from Sigma-Aldrich, target ID MM0000389854). Transduced cells were single cell sorted for RFP signal using FACS. Disruption of *Abcb6* gene was determined by the absence of ABCB6 protein using immunoblotting. These *Abcb6-null* MEL cells were than transduced with a retrovirus expressing either Flag-tagged ABCB6 WT-IRES-GFP or A492T-IRES-GFP. Transduced cells were FACS sorted twice for GFP to obtain cells that expressed similar levels of GFP.

### Preparation of MEL crude membrane vesicles

Cells were pelleted and washed with 1 × Hank's buffered saline before suspension in hypotonic buffer (0.5 mM Tris (pH 7.5) and 0.1 mM EDTA). Cells were lysed by one freeze–thaw cycle and then resuspended in hypotonic buffer followed by centrifugation in a MLS50 swinging bucket rotor at 100,000*g*. The resulting pellet was then resuspended into homogenization buffer (250 mM sucrose, 50 mM Tris and 250 mM CaCl_2_, (pH 7.5)) and homogenized with a tight dounce pestle. After centrifugation, the pellets were resuspended in homogenization buffer and re-homogenized. The supernatants of each spin were pooled and layered over a 35% sucrose pad formed in 50 mM Tris (pH 7.5) After centrifugation 100,000*g* (4 °C), the interface was carefully removed and diluted with 50 mM Tris, (pH 7.5) and 25 mM sucrose. The crude membranes were pelleted at 100,000*g* in the MLS50 rotor and then resuspended in ghost lysis buffer (50 mM Tris (pH 7.5) followed by passage through a 27 G needle. These crude membrane vesicles were used in the porphyrin transport assay described below.

### Porphyrin transport assay

Transport in red cell ghosts was determined after preparation of red cell ghosts from peripheral blood samples collected from adult C57BL/6 WT, *Abcb6*^−*/*−^ or *Abcg2KO*^−*/*−^ (gift from Dr Sorrentino's lab, SJCRH) mice. The blood was centrifuged at 10,000*g* to separate blood cells from plasma. The resulting blood cell pellet was washed with ice-cold Ghost Wash buffer (150 mM NaCl and 10 mM Tris (pH=7.4)). These cells were lysed by suspension in 10 mM Tris (pH=7.4)) and the lysate collected by centrifugation at 21,200*g*. These steps were repeated until the supernatant was clear. This pelleted fraction was then repeatedly washed in 10 mM Tris (pH=7.4)) until it became translucent and was devoid of red colour. These red cell ghosts were then incubated overnight at 4 °C followed by passage through a 27 G needle to form everted membranes. The ATP-dependent porphyrin transport assays were performed as previously described using either 2 mM ATP or AMP-PNP with an ATP-regenerating system^18^. Porphyrins transported into the ghosts were measured by fluorescence (Ex 405 nm/Em 618 nm).

### Cytokine measurement

Serum cytokines from adult female mice were measured using the enzyme-linked immunosorbent assay-based MilliplexMAP mouse cytokine/chemokine 96-well plate assay according to the manufacturer's procedures (Millipore, Billerica, MA).

### Statistical analysis

Fisher's exact test and Student's *t*-test analyses were performed using GraphPad Prism 5 (GraphPad Software, San Diego, CA).

### Data availability

Exome-sequencing data that support the findings of this study have been deposited in European Nucleotide Archive with the study accession code PRJEB14229 (http://www.ebi.ac.uk/ena/data/view/PRJEB14229). The authors declare that other data supporting the findings of this study are available within the article and its Supplementary Information files or from the authors on request.

## Additional information

**How to cite this article:** Fukuda, Y. *et al.* The severity of hereditary porphyria is modulated by the porphyrin exporter and Lan antigen ABCB6. *Nat. Commun.* 7:12353 doi: 10.1038/ncomms12353 (2016).

## Supplementary Material

Supplementary Information Supplementary Figures 1-7 and Supplementary Tables 1-5

## Figures and Tables

**Table 1:** Non-synonymous *ABCB6* variants identified in the cohort.

Haem synthesis defect	Number of patients	*ABCB6* non-synonymous variant (number detected)	% MAF (European American)	NCBI dbSNP ID
*HMBS*	18	A492T (1)	0.907	rs147445258
		G588S (2)	0.663	rs145526996
		A681T (1)	0.081	rs142421126
*CPOX*	10	R192Q (1*)	0.419	rs150221689
*PPOX*	7	R276W (2)	1.327	rs57467915
*FECH*	1	T521S (1)	0.395	rs149363094
Total	36	8		

MAF, minor allele frequency; NCBI, National Center for Biotechnology Information.

Variant information and their MAF in European Americans are summarized.

^*^Asymptomatic patient.
