# Supplementary material for: The severity of hereditary porphyria is modulated by the porphyrin exporter and Lan antigen ABCB6
Source: Nat Commun. 2016 Aug 10;7:12353. doi: 10.1038/ncomms12353 (PMC4987512; doi:10.1038/ncomms12353)
Supplement: Supplementary Information — Supplementary Figures 1-7 and Supplementary Tables 1-5 [file ncomms12353-s1.pdf]

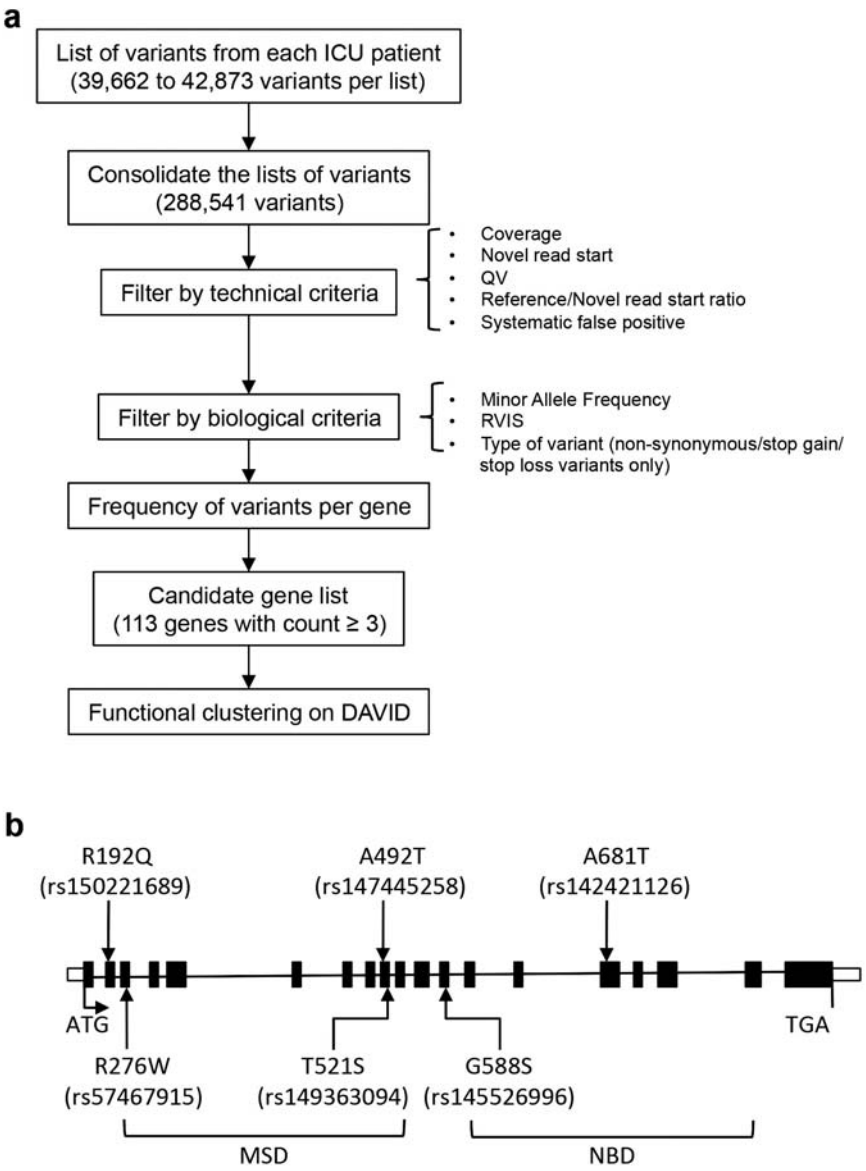

Supplementary Figure 1. Filtering strategy in candidate gene analysis and exon position of ABCB6 variants found in the cohort. a) Flow chart describing the exome sequencing data analysis of Symptomatic-ICU patients. **b**) Schematic drawing of *ABCB6* gene is shown with *ABCB6* non-synonymous variants indicated with arrows. MSD, membrane-spanning domain; NBD, nucleotide-binding domain.

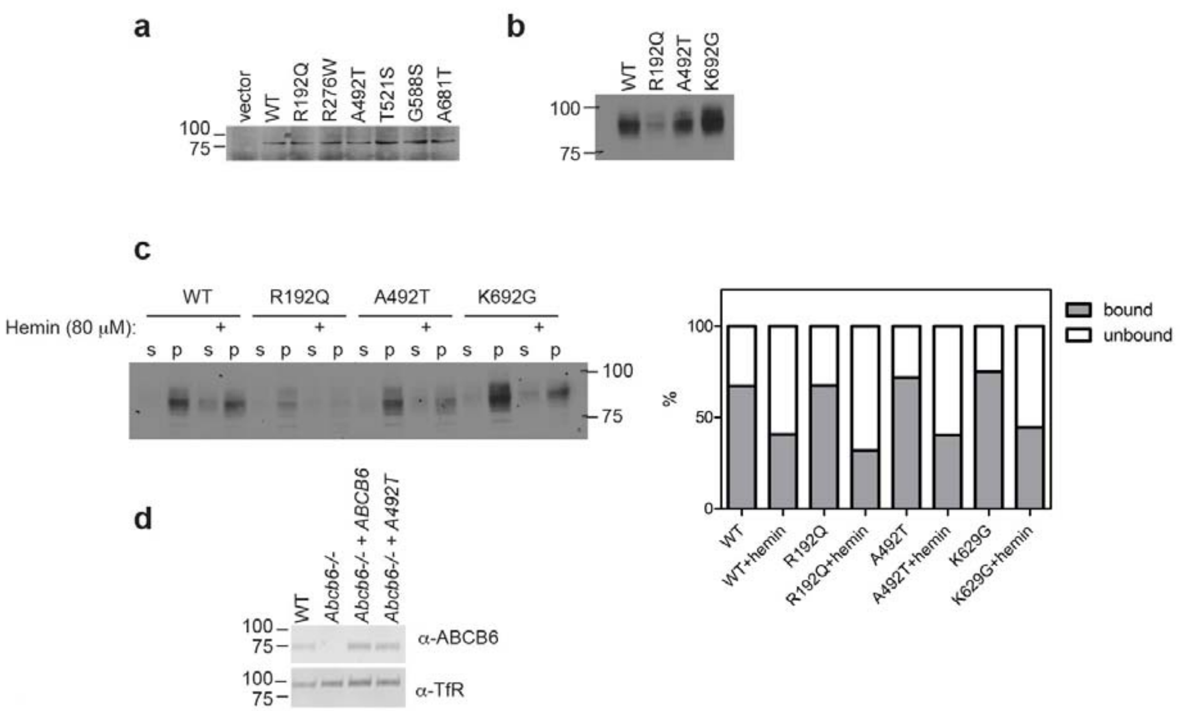

Supplementary Figure 2. ABCB6 variant alleles do not have altered transcription/translation and heme binding. a) Immunoblot of *in vitro* transcription/translation-coupled assay using plasmids encoding listed variant ABCB6 using anti-FLAG antibody. NIH3T3 cells were transiently transfected with plasmids encoding WT, R192Q, A492T, or K629G-V5. Immunoblots of **b)** total lysate (input) and **c)** hemin-agarose pull-down assays in the presence (+) or absence of heme to determine heme-binding capacity of ABCB6 variants (bound (p, pellet) and unbound (s, soluble)). **d)** Membrane vesicle preparations from indicated MEL cell lines were analyzed for ABCB6 expression (TfR, transferrin receptor is shown as a control).

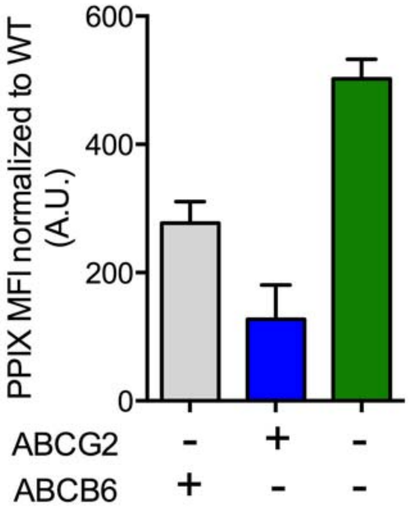

Supplementary Figure 3. Both ABCB6 and ABCG2 contribute to PPIX efflux in mouse reticulocytes. Peripheral blood collected from WT or *Abcb6*$^{-/-}$ mice were treated with either DMSO or 10 μM PPIX with or without 5 μM FTC as indicated for 1 hr at 37°C. Intracellular PPIX concentrations in reticulocytes (TO^{+}/Ter119^{+}) were measured by FACS. PPIX levels were normalized to WT cells incubated with PPIX (Abcg2 was inhibited by FTC, while *Abcb6* was genetically knocked out). A.U., arbitrary unit.

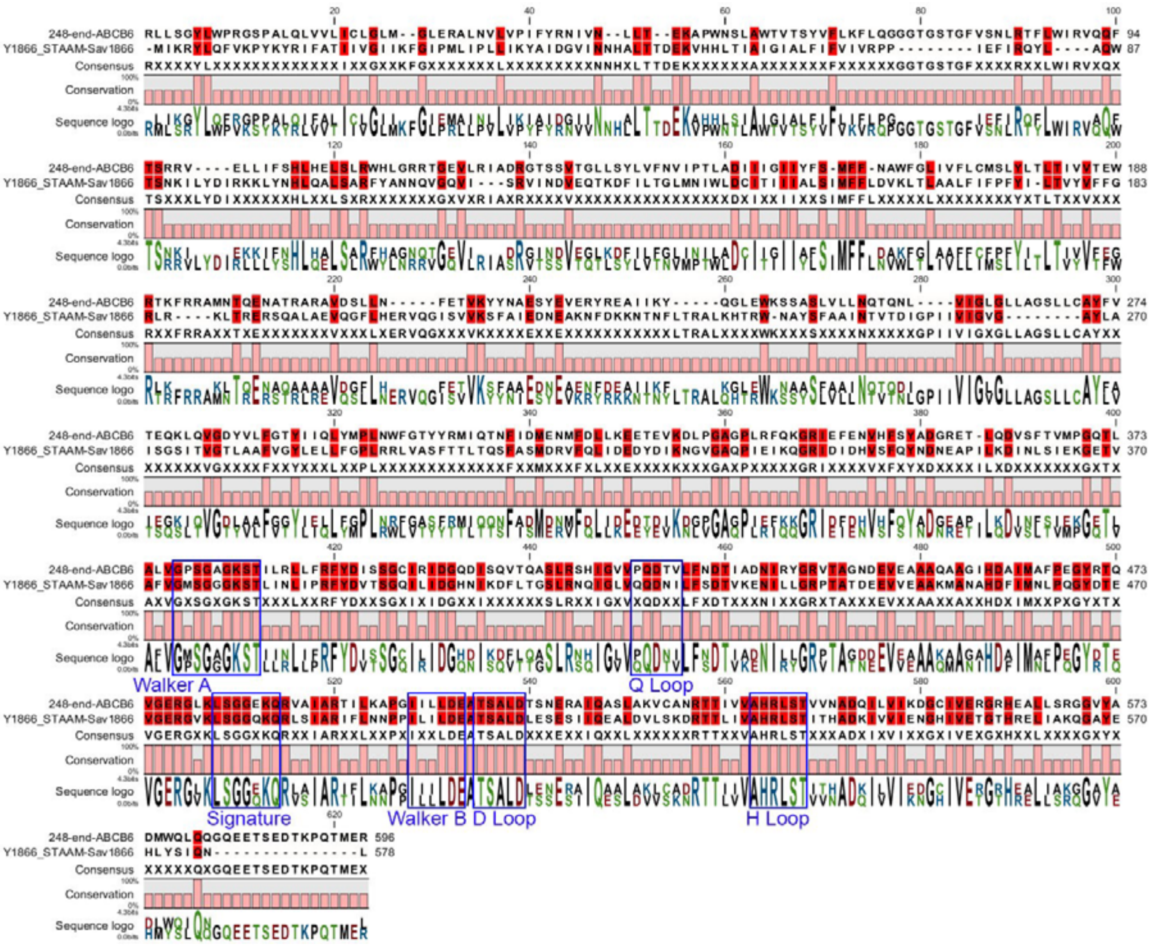

Supplementary Figure 4. Amino acid sequence alignment of human ABCB6 and Sav1866. The sequence alignment of ABCB6 (starting from aa 248) and Sav1866 was performed using CLC Bio software (Qiagen). Identical residues are boxed in red, and blue boxes highlight conserved motifs in the nucleotide-binding domain.

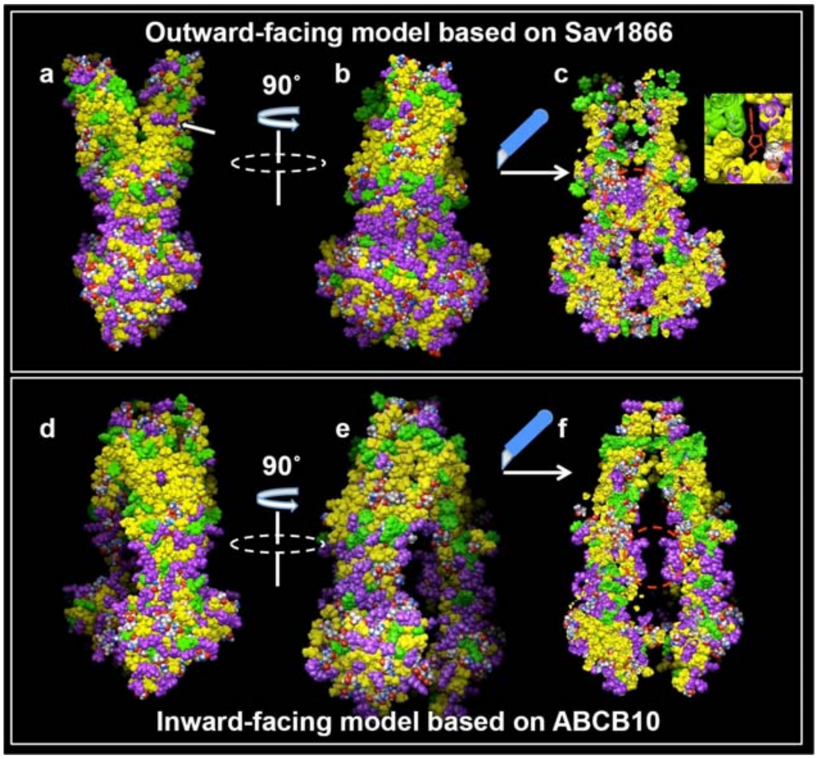

Supplementary Figure 5. Views of the outward-facing (a-c) and inward facing (d-f) homology models for ABCB6. Surface views (a,b & d,e) are orthogonal to each other, as indicated by the rotation symbol. Central slices (c,f) are shown where the slice is taken orthogonal to the viewing direction in (b,e). Color key for amino acid residue types: Yellow – hydrophobic. Green-aromatic. Purple-charged. CPK- polar. The lower intracytoplasmic loops and nucleotide-binding domains display surface residues that are predominantly polar and charged, but with hydrophobic cores that are revealed by the central slices (c,f). The cluster of charged residues in the central translocation channel is intriguing (red dashed circle, views c & f). The upper transmembrane segment is visible as a 4 nm wide band of hydrophobic residues bounded by clusters of aromatic residues. A few surface-exposed charged residues in the TM region (a,b,d,e) are visible and will be energetically unfavored, however there is no obvious patch of polar/charged residues that might indicate an interface for the additional N-terminal transmembrane helices of ABCB6 (which cannot be modeled). The charged pair R276/E275 discussed in the main text is indicated by the white arrow (view a). Note this pair is mostly internalized in the inward-facing model (view d).
Inset, view c: No ADP nor ATP was employed in the homology modeling. Nevertheless the ATP binding cavity is preserved in the outward-facing model, including a stacking interaction with a Tyr residue. The small cavity where the ATP terminal phosphate is bound is also preserved (this phosphate is lacking in the Sav1866 model which is the ADP bound state) and also its exit channel to the surface is preserved. This implies that this model – even after significant energy minimization is reasonable.

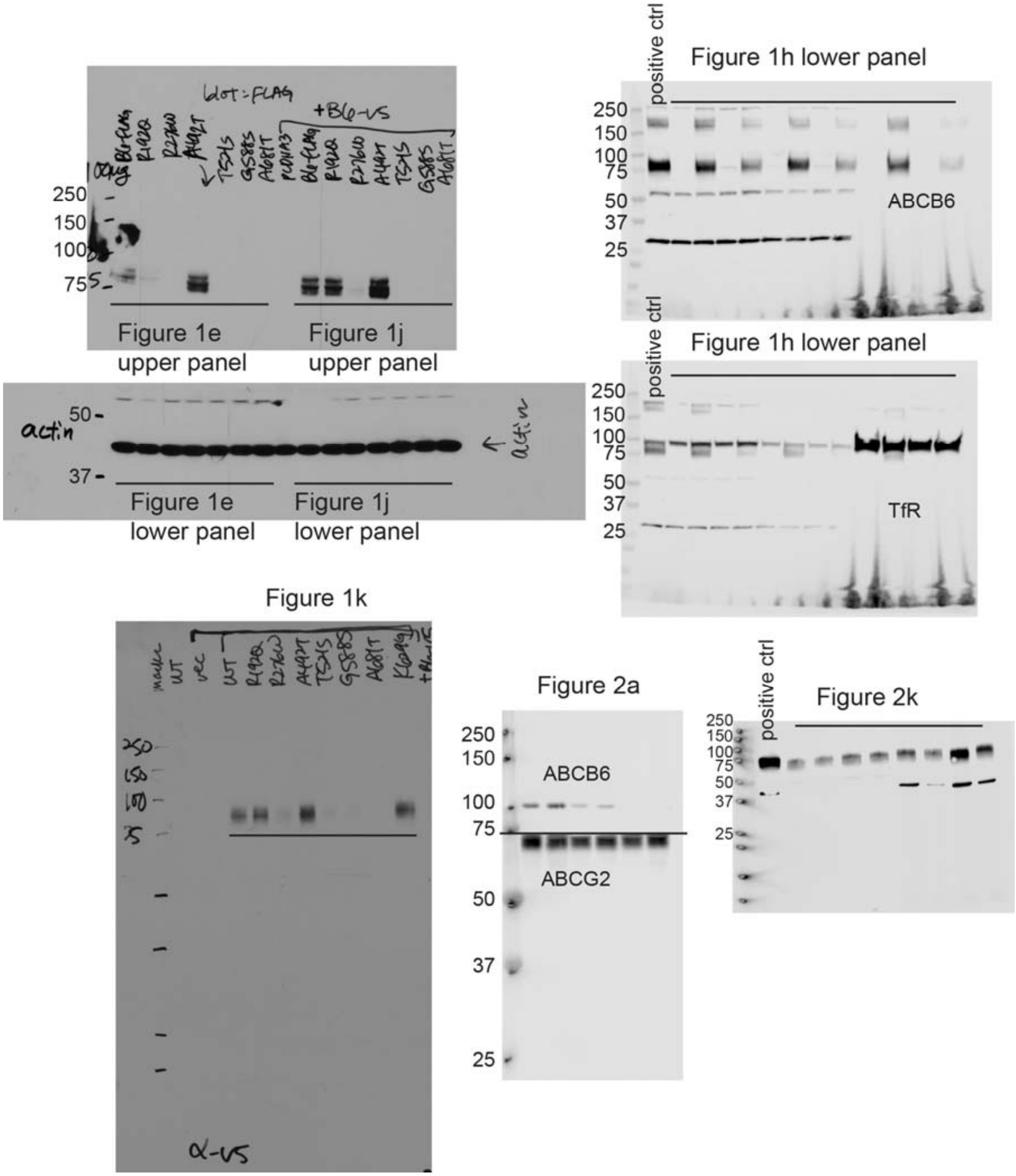

Supplementary Figure 6. Full scan of immunoblots in main figures. Immunoblots from Figures 1e, 1h, 1j, 1k, 2a, 2k are shown.

Supplementary Figure 2a

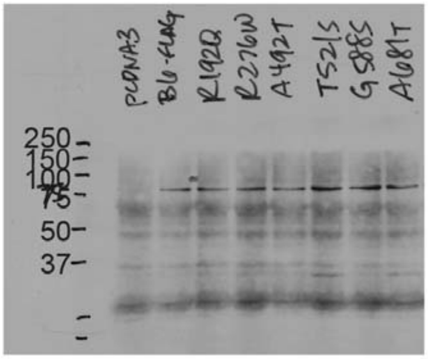

Supplementary Figure 2b, 2c

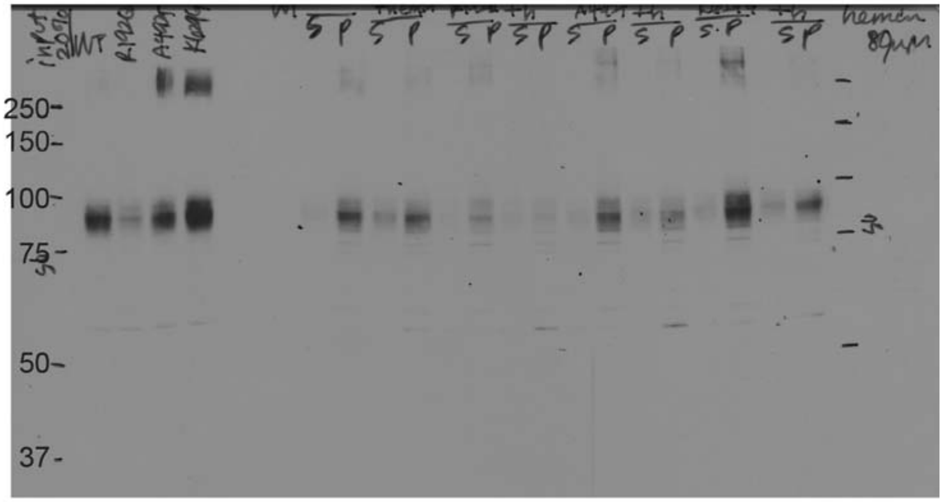

Supplementary Figure 2d

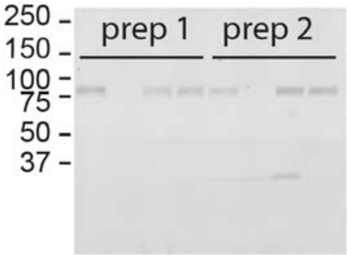

Supplementary Figure 7. Full scan of immunoblots in supplementary figures. Immunoblots from Supplementary Figures 2a, 2b, 2c, and 2d are shown.

Table 1. Detailed patient information. Patients within the same family are preceded by the same letter in the sample ID. In addition to a nonsense *HMBS* mutation, exome sequencing discovered that patient C1 was heterozygous for a FECH p.G55C mutation. ##Further study showed that she was also heterozygous for the IVS3-48T>C splicing mutation known to cause erythropoietic protoporphyria when *in trans* with a FECH mutation. Family studies were not available to determine the phase between the two *FECH* mutations. ###Patient X1 inherited an atypical low expression allele that is not the commonly described IVS3-48T>C splicing mutation. This low expression allele has 48% of cDNA compared to the normal allele and is *in trans* with FECH p.F260L. Sx, symptomatic.

Sex	ID #	Diagnosis	Porphyria mutation	ABCG2 variant	ABCB6 variant	Phenotypic classification	Porphyrin Cr^{-1}(0–35 nmol mmol^{-1})
M	A1	AIP	HMBS p.G24D	nil	nil	Sx-no admission	**73**
F	A2	AIP	HMBS p.G24D	nil	nil	Sx-no admission	17
F	B1	AIP	HMBS c.33+1G>T	nil	nil	Sx-admission	na
F	B2	AIP	HMBS c.33+1G>T	p.V12M	nil	Asymptomatic	na
F	B3	AIP	HMBS c.33+1G>T	p.V12M	nil	Asymptomatic	na
F	B4	AIP	HMBS c.33+1G>T	nil	nil	Sx-admission	**106**
M	B5	AIP	HMBS c.33+1G>T	nil	nil	Sx-admission	na
F	B6	AIP	HMBS c.33+1G>T	nil	p.G588S	Sx-admission	**346**
F	B7	AIP	HMBS c.33+1G>T	p.V12M	p.G588S	Sx-ICU	**61**
F	C1	AIP	HMBS p.G111*; FECH p.G55C and FECH IVS3-48T>C ##	nil	nil	Sx-ICU	**328**
F	D1	AIP	HMBS c.345-1G>A	nil	nil	Sx-admission	**236**
F	E1	AIP	HMBS p.R116W	p.V12M	nil	Sx-no admission	na
F	F1	AIP	HMBS p.R149*	nil	p.A681T	Sx-ICU	**218**
M	G1	AIP	HMBS p.R195H	nil	nil	Asymptomatic	**51**
F	H1	AIP	HMBS p.R225*	nil	nil	Sx-no admission	na
F	I1	AIP	HMBS p.R225*	p.V12M	p.A492T	Sx-ICU	**169**
F	J1	AIP	HMBS c.913-2A>G	p.V12M	nil	Sx-ICU	na (overseas)
F	K1	AIP	HMBS p.Q314Vfs*8	nil	nil	Sx-admission	**35-438**
M	L1	HCP	CPOX p.P134H	nil	nil	Asymptomatic	na
F	M1	HCP	CPOX p.T286K	p.V12M	nil	Sx-admission	11
F	M2	HCP	CPOX p.T286K	p.V12M	nil	Sx-admission	**55**
F	N1	HCP	CPOX p.A293P	nil	nil	Sx-no admission	**86**
M	N2	HCP	CPOX p.A293P	nil	p.R192Q	Asymptomatic	9
F	O1	HCP	CPOX p.R332Q	nil	nil	Sx-admission	**49**
F	P1	HCP	CPOX p.Q355P	nil	nil	Asymptomatic	**206**
F	P2	HCP	CPOX p.Q355P	nil	nil	Sx-no admission	na
M	P3	HCP	CPOX p.Q355P	nil	nil	Asymptomatic	na
F	Q1	HCP	CPOX p.H431Lfs*60	nil	nil	Asymptomatic	Na
F	R1	VP	PPOX p.R59W	nil	nil	Asymptomatic	8
F	S1	VP	PPOX p.R59W	nil	nil	Asymptomatic	29
M	T1	VP	PPOX c.471+1G>A	nil	p.R276W	Sx-ICU	**621**
F	U1	VP	PPOX p.Q189*	nil	nil	Sx-no admission	23
F	V1	VP	PPOX p.Q375*	p.V12M	nil	Sx-no admission	**272**
F	W1	VP	PPOX p.E378Rfs*24	nil	p.R276W	Sx-no admission	**45**
F	W2	VP	PPOX p.E378Rfs*24	nil	nil	Sx-admission	8
F	X1	EPP	FECH p.F260L ###	p.V12M	p.T521S	Sx-ICU, deceased	**181**

Supplementary Table 2. Candidate genes used for analysis on DAVID and their respective RVIS score. A higher RVIS score means the gene is more tolerant to variants.

Gene	RVIS
HMCN1	-3.69
COL5A1	-3.01
CELSR1	-2.82
FRY	-2.66
NCOR2	-2.60
MLL3	-2.52
ABCA7	-2.15
CACNA1H	-2.06
LAMA3	-2.06
HTT	-2.03
IFT172	-1.96
UTRN	-1.55
RANBP2	-1.54
DCHS1	-1.38
IGSF3	-1.34
ATP12A	-1.32
DYSF	-1.31
SIPA1L2	-1.31
OSBPL6	-1.30
DEPDC5	-1.12
DHX34	-1.02
CHD6	-0.97
TTC3	-0.97
MYH7B	-0.89
CHPF	-0.86
ACOX3	-0.79
ADAMTS20	-0.75
MYH8	-0.69
TMC1	-0.64
MYH13	-0.64
ENGASE	-0.63
PLCG2	-0.60
GOLGA3	-0.57
RGS12	-0.46
GTPBP2	-0.43
MST1	-0.42
FOXK2	-0.42
NCAPD3	-0.37

Gene	RVIS
MYO5B	-0.37
ABCB6	-0.37
HMBS	-0.34
GOLIM4	-0.33
PCM1	-0.32
HSPG2	-0.31
KRT6B	-0.30
FAAH2	-0.29
DHX58	-0.28
ZFYVE26	-0.27
PAQR6	-0.27
ANO9	-0.26
FRG1	-0.25
NOL6	-0.23
GPCPD1	-0.20
ABCG2	-0.20
C16orf7	-0.18
SYNE1	-0.16
PPEF1	-0.16
SETX	-0.14
RBM23	-0.13
CDC27	-0.12
OTOF	-0.11
GGT1	-0.09
ZNF18	-0.07
HABP2	-0.06
NTRK1	-0.06
MCM10	-0.03
TMCO7	-0.01
TBC1D1	0.01
TIMM44	0.09
ATRN	0.12
ITIH2	0.15
ADRA1A	0.16
DNAJC13	0.17
TCTN1	0.18
GAS8	0.21
PTPRC	0.23

Gene	RVIS
SSFA2	0.24
DNPEP	0.24
PUS3	0.24
ZNF750	0.32
TCERG1L	0.33
RP2	0.35
GRAMD2	0.35
PKHD1	0.38
IQGAP2	0.39
DLG1	0.40
FAM104B	0.41
SCYL1	0.47
SORBS1	0.48
MRPS27	0.49
MORC1	0.49
TNFRSF13B	0.53
AHCTF1	0.54
FSTL5	0.54
LETM1	0.54
SMPDL3A	0.57
ADAM18	0.59
TMEM123	0.59
DEFB104A	0.61
SDSL	0.62
PKP2	0.65
CCDC135	0.72
ABCA4	0.74
OR5V1	0.75
CARKD	0.76
CLYBL	0.77
B4GALNT2	0.80
NEB	0.88
GAS2L3	0.89
ZNF763	0.93
N4BP2	0.95
TAS2R19	1.00
RENBP	1.00

Supplementary Table 3. List of genes in annotation cluster1 (highest enrichment score) from DAVID. Enrichment score is calculated from the minus log transformation of the geometric mean of all P-values in the cluster. Benjamini: P-value corrected for multiple testing using the Benjamini and Hochberg method. A total of 35 annotation clusters were generated. GOTERM_MF_FAT and GOTERM_CC_FAT: Gene Ontology (GO) term >Molecular Function and >Cellular Component on the GO database, respectively.

Annotation cluster 1				
Category	Term	Genes	*P*-Value	Benjamini
GOTERM_MF-FAT	GO:0030554~adenyl nucleotide binding	ABCA7, RP2, ATP12A, MORC1, ABCA4, ABCB6, TIMM44, MYH8, ABCG2, ACOX3, SETX, N4BP2, RENBP, SCYL1, NTRK1, DHX34, MYH13, CHD6, MYH7B, MYO5B, DHX58	0.0014	0.10
GOTERM_MF-FAT	GO:0001883~purine nucleoside binding		0.0017	0.08
GOTERM_MF-FAT	GO:0001882~ nucleoside binding		0.0018	0.07

Supplementary Table 4. Genotype frequency of ABCB6 variants that result in non-synonymous amino acid changes. Data extracted from EVS database in September 2013. All the variants were present as heterozygous. There were total of 419 variant alleles found in at least 4602 individuals.

Protein Change	cDNA Change	EA Genotype #
p.(P837L)	c.2510C>T	AA=0/AG=1/GG=4299
p.(Y818C)	c.2453A>G	CC=0/CT=1/TT=4299
p.(A810V)	c.2429C>T	AA=0/AG=0/GG=4300
p.(G800V)	c.2399G>T	AA=0/AC=1/CC=4299
p.(T778I)	c.2333C>T	AA=0/AG=0/GG=4300
p.(R776S)	c.2326C>A	TT=0/TG=1/GG=4299
p.(V772F)	c.2314G>T	AA=0/AC=1/CC=4299
p.(R739C)	c.2215C>T	AA=0/AG=4/GG=4296
p.(G730R)	c.2188G>A	TT=0/TC=0/CC=4300
p.(R723Q)	c.2168G>A	TT=0/TC=9/CC=4291
p.(R723W)	c.2167C>T	AA=0/AG=1/GG=4299
p.(I705M)	c.2115C>G	CC=0/CG=0/GG=4300
p.(A702V)	c.2105C>T	AA=0/AG=0/GG=4300
p.(R685C)	c.2053C>T	AA=0/AG=0/GG=4300
p.(D682N)	c.2044G>A	TT=0/TC=0/CC=4300
p.(A681T)	c.2041G>A	TT=0/TC=7/CC=4293
p.(D672E)	c.2016C>G	CC=0/CG=1/GG=4299
p.(D672N)	c.2014G>A	TT=0/TC=1/CC=4299
p.(A660V)	c.1979C>T	AA=0/AG=0/GG=4300
p.(R648Q)	c.1943G>A	TT=0/TC=1/CC=4299
p.(V609M)	c.1825G>A	TT=0/TC=0/CC=4300
p.(E604D)	c.1812G>C	GG=0/GC=1/CC=4299
p.(G588S)	c.1762G>A	TT=0/TC=57/CC=4243
p.(R584H)	c.1751G>A	TT=0/TC=1/CC=4299

p.(L577F)	c.1729C>T	AA=0/AG=1/GG=4299
p.(F565S)	c.1694T>C	GG=0/GA=1/AA=4299
p.(P543L)	c.1628C>T	AA=0/AG=1/GG=4299
p.(T521S)	c.1562C>G	CC=0/CG=34/GG=4266
p.(T521A)	c.1561A>G	CC=0/CT=2/TT=4298
p.(S513P)	c.1537T>C	GG=0/GA=0/AA=4300
p.(G512S)	c.1534G>A	TT=0/TC=0/CC=4300
p.(A511T)	c.1531G>A	TT=0/TC=1/CC=4299
p.(A492T)	c.1474G>A	TT=0/TC=78/CC=4222
p.(R475C)	c.1423C>T	AA=0/AG=0/GG=4300
p.(L457P)	c.1370T>C	GG=0/GA=1/AA=4299
p.(V454A)	c.1361T>C	GG=0/GA=2/AA=4298
p.(N447S)	c.1340A>G	CC=0/CT=1/TT=4299
p.(I429V)	c.1285A>G	CC=0/CT=2/TT=4298
p.(L425V)	c.1273C>G	CC=0/CG=0/GG=4300
p.(L415F)	c.1243C>T	AA=0/AG=1/GG=4299
p.(R371Q)	c.1112G>A	TT=0/TC=1/CC=4297
p.(L358V)	c.1072C>G	CC=0/CG=1/GG=4295
p.(R343Q)	c.1028G>A	TT=0/TC=10/CC=4286
p.(F332L)	c.994T>C	GG=0/GA=0/AA=4294
p.(K313E)	c.937A>G	CC=0/CT=0/TT=4300
p.(T307S)	c.920C>G	CC=0/CG=1/GG=4299
p.(L302V)	c.904C>G	CC=0/CG=0/GG=4300
p.(P283S)	c.847C>T	AA=0/AG=0/GG=4286
p.(R276W)	c.826C>T	AA=0/AG=114/GG=4183
p.(L263V)	c.787C>G	CC=0/CG=1/GG=4298
p.(S228R)	c.684C>G	CC=0/CG=1/GG=4299
p.(E220V)	c.659A>T	AA=0/AT=6/TT=4294

p.(R210C)	c.628C>T	AA=0/AG=1/GG=4299
p.(G208R)	c.622G>A	TT=0/TC=1/CC=4299
p.(G197R)	c.589G>A	TT=0/TC=1/CC=4299
p.(R192Q)	c.575G>A	TT=0/TC=36/CC=4264
p.(R192W)	c.574C>T	AA=0/AG=21/GG=4279
p.(A164T)	c.490G>A	TT=0/TC=3/CC=4297
p.(A157V)	c.470C>T	AA=0/AG=1/GG=4299
p.(S147G)	c.439A>G	CC=0/CT=2/TT=4298
p.(R129Q)	c.386G>A	TT=0/TC=2/CC=4297
p.(L125F)	c.373C>T	AA=0/AG=0/GG=4292
p.(A92V)	c.275C>T	AA=0/AG=3/GG=4229

Supplementary Table 5. Predicted effect of rare ABCB6 variants identified in porphyric patients. Different *in silico* protein prediction methods were used (stability, conservation, etc.)

ABCB6	BLOSUM50	PAM250	Grantham Value	PolyPhen-2 (HumDiv class:Score)	SIFT	I-Mutant Suite
**Threshold**	<0	<0	>50	>0.85	<0.05	<0.5; >0.5
**R192Q**	1	1	43	probably-damaging:0.985	0.01	Decrease, -0.86
**R276W**	-3	-2	101	probably-damaging:1.0	0.00	Decrease -0.36
**A492T**	0	1	58	probably-damaging:0.979	0.07	Decrease -0.49
**T521S**	2	1	58	benign:0.0	0.41	Decrease -0.65
**G588S**	0	1	56	probably-damaging:1.0	0.00	Decrease -1.21
**A681T**	-1	-1	58	possibly-damaging:0.941	0.40	Decrease -1.08
